# Meta-Analysis of the Potential Role of miRNA-21 in Cardiovascular System Function Monitoring

**DOI:** 10.1155/2020/4525410

**Published:** 2020-03-31

**Authors:** Olga Krzywińska, Marietta Bracha, Caroline Jeanniere, Emeline Recchia, Kornelia Kędziora Kornatowska, Mariusz Kozakiewicz

**Affiliations:** ^1^Department of Geriatrics, Division of Biochemistry and Biogerontology, L. Rydygier Collegium Medicum in Bydgoszcz, Nicolaus Copernicus University in Torun, Poland; ^2^School of Engineering-Biology and Health Systems, University of Angers-Polytech Angers, France; ^3^Department of Geriatrics, L. Rydygier Collegium Medicum in Bydgoszcz, Nicolaus Copernicus University in Torun, Poland

## Abstract

MicroRNAs (miRNAs) are short and noncoding RNA fragments that bind to the messenger RNA. They have different roles in many physiological or pathological processes. MicroRNA-21, one of the first miRNAs discovered, is encoded by the MIR21 gene and is located on the chromosomal positive strand 17q23.2. MicroRNA-21 is transcribed by polymerase II and has its own promoter sequence, although it is in an intron. It is intra- and extracellular and can be found in many body fluids, alone or combined with another molecule. It regulates many signalling pathways and therefore plays an important role in the cardiovascular system. Indeed, it is involved in the differentiation and migration of endothelial cells and angiogenesis. It contributes to the reconstruction of a myocardial infarction, and it can also act as a cellular connector or as an antagonist to cardiac cell apoptosis. By playing all these roles, it can be interesting to use it as a biomarker, especially for cardiovascular diseases.

## 1. Introduction

The discovery of the *lin-4* gene coding small RNA [1] and the discovery of *let-7* RNA in the nematode *C. elegans* [2] interested molecular and cellular biologists but also clinicians. Further studies addressing the former confirmed the occurrence of genes encoding short RNA sequences, and this led to the establishment of a mechanism for regulating RNA interference gene expression. This discovery has been recognized by the scientific community and awarded the Nobel Prize in 2006 [3].

RNA fragments known as microRNA (miRNA) are short (approx. 22 nucleotides), endogenous, noncoding fragments of ribonucleic acid, which bind the messenger RNA (mRNA) causing its degradation or inhibition of translation and consecutively regulate gene expression at the posttranscriptional level [4]. miRNA molecules play a role in many physiological and pathological processes. These processes include cell proliferation and differentiation, including differentiation of haematopoietic stem cells; apoptosis; haematopoiesis including erythropoiesis; megakaryocytosis; differentiation of skeletal muscle cells; embryogenesis, neurogenesis; angiogenesis; exocytosis; insulin secretion regulation; differentiation of adipocytes and mononuclear cells, immune processes; and inflammatory conditions [5–9]. The multifunctionality of these small molecules has been extensively investigated in cancers, viral and bacterial infections, neurological diseases, and cardiovascular diseases. However, the miRNA expression varies depending on the type of tissue and cell, as well as its metabolism and the pathophysiological changes accompanying the disease process [10]. Until 2014, more than 2000 different sequences of miRNA were described and cataloged in miRBase [11]. The most recent data indicate that the human genome contains 1917 precursors and 2654 mature microRNA sequences [12]. Current research focuses on defining the role of microRNA in the pathomechanisms of various diseases and their use in clinical practice.

Cardiovascular diseases (CVD) are among the most common diseases of modern societies, and they are also, similar to cancers, one of the main causes of mortality in the world (Heart Disease and Stroke Statistics—2019 Update). Identifying factors that can significantly reduce patient mortality rates remains a challenge. A more effective prevention and early diagnosis of acute coronary syndrome (ACS), whose clinical presentations are myocardial infarction (MI) and unstable angina (UA), may significantly decrease the occurrence of heart failure (HF) and thus reduce the cardiovascular mortality rate.

Previous scientific reports confirm the role of miRNA in the pathogenesis of cardiovascular diseases. This article focuses on one specific microRNA—miR-21—and attempts at gathering knowledge about its basic biology and the underlying function of the pathology of the cardiovascular system.

### 1.1. Biogenesis

**Figure 1:**
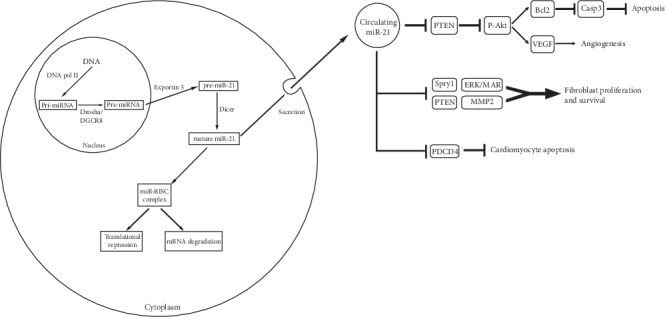
Biogenesis (a) and main pathways (b) of miRNA-21. (a) MiR-21 biogenesis looks similar to that of other miRNA molecules: pri-miR-21 is processed by Drosha endonuclease and DGCR8 protein to pre-miR-21 (this is done in the cell nucleus); then, pre-miR-21 is transferred to the cytoplasm by the enzyme exportin 5. In the cytoplasm, the Dicer enzyme removes the final loop of the pre-miR-21 structure to form a mature miR-21. (b) Mature miR-21 can act intracellularly by silencing mRNA, or it can be secreted outside the cell and regulate among others inhibition of apoptosis, induction of angiogenesis, and proliferation and survival of fibroblasts.

Most miRNA genes are located between protein-coding sequences as standalone transcriptional units or in coding sequences or are located in introns [13]. MicroRNA-21 also known as hsa-miR-21 (or miRNA21 or miR-21), which is one of the first identified human microRNAs, is encoded by the gene MIR21 [14]. The microRNA-21 gene is located on the positive chromosome strand 17q23.2 within the intron gene TMEM49 region coding the transmembrane protein 49 (TMEM49) (also known as protein with human vacuolar membrane 1 (VMP1)) [15]. Although miR-21 is an intron microRNA, the original miR-21 transcript—the PRI-miR-21—is independently transcribed by polymerase II. Furthermore, it has its own promotor regions and is terminated by its own tail poli(A) [16].  Figure 1 shows the main pathways of metabolism as well as biogenesis of miRNA-21.

The original transcript for MIR-21 has a length of 3433 nucleotides. Pri-mi-RNA is processed by the Drosha endonuclease (RNase III enzyme) and DGCR8 (dsRNA binding protein) to the precursor of the pre-miRNA parent loop in the nucleated cell [17]. The precursor MIR-21 has a length of 72 alkalis (pre-MIR-21), which creates a secondary structure and contains mature miRNA sequences, and is then transferred from the nucleus to the cytoplasm by the enzyme exportin 5 [18]. In the cytoplasm, the second enzyme RNase III, Dicer, removes the final loop of the pre-MI-RNA structure, generating approximately 20-nucleotide duplexes, in which one strand is degraded and the other is incorporated into the protein complex, the RNA-induced silencing complex (RISC), a partially complementary target for mRNA [19]. A mature single-stranded MIR-21 has a length of 22 nucleotides (sequence: UAGCUUAUCAGACUGAUGUUGA—Atlas of Genetics and Cytogenetics).

### 1.2. Secretion and Circulation

MicroRNA can be located intracellularly or can be actively secreted by cells and contribute to intercellular or cellular communication [20]. So far, circulating microRNA has been reported in whole blood, peripheral blood mononuclear cells (PBMCs), platelets, serum, plasma, and other body fluids such as urine, breast milk, saliva, and tears [21, 22]. Despite the presence of extracellular RNase, miRNA remains in a stable form in the blood [23]. MicroRNA can be secreted from cells in the exosomes, by combining it with a protein complex (proteins in the Argonaute family), by integrating it into a complex of lipoproteins (HDL), through apoptotic bodies, or in microbubbles by interacting with the membrane proteins or circulating as free miRNA by natural leakage from the cell or as a product of dead cells [24]. Thanks to membrane vesicles and protein/lipoprotein complexes, miRNA circulatory effects remain stable and can be useful as easily available biomarkers.

### 1.3. miR-21 in the Cardiovascular System

MiR-21 plays a key role in many biological processes including those in the cardiovascular system. Like all miRNAs, miR-21 is regulated at the posttranscriptional level by various regulatory proteins including the TGF-*β* receptor (TGF-*β*R), the phosphatase and tensin homolog (PTEN), and Smad7 [25].

MiR-21 is one of the microRNAs with a very high expression in endothelial cells, which is why it participates in the differentiation and migration of endothelial cells and angiogenesis [26]. Di Bernardini and coauthors explained the signalling pathway of endothelial differentiation from induced pluripotent stem cells in functional endothelial cells, which is regulated by microRNA-21 (via the PTEN-Akt pathway) and transformative growth factor beta2 (TGF-*β*2) [27]. During studies on the effects of resveratrol on the behavior of resident vascular stem cells, it was confirmed that miR-21 and the PTEN/akt/*β*-catenin pathway are involved in the direction of stem/progenitor cell differentiation towards the endothelial line [28].

Recent scientific reports also confirm the specific role of miR-21 in the regulation of the biological function of progenitor endothelial cells (EPC). Studies have shown that exosomes from endothelial progenitor cells facilitate the repair of vascular endothelial cells by transporting miR-21-5p, which specifically suppresses the expression of the thrombospondin 1 (THBS1) angiogenesis inhibitor in endothelium cells [29]. It was also found that miR-21 may bind membrane-related FASLG protein by inhibiting its expression and ultimately promoting EPC proliferation and angiogenesis [30].

The role of miR-21 as a Sprouty1 inhibitor (SPRY1) has been described, which can regulate the activation of ERK and RhoB kinases, key cytoskeletal regulators, affecting inter alia the migration of endothelial cells and their angiogenic properties [31]. On the other hand, Liu and coauthors showed that miR-21 induces angiogenesis by targeting PTEN, leading to the activation of AKT and ERK1/2 signalling pathways, thereby increasing the expression of HIF-1*α* and VEGF, which is the key objective of miR-21 in regulating angiogenesis [32].

Zhu and coauthors revealed that microRNA-21 expression increases with age, resulting in lower Hmga2 levels, which in turn activates p16^Ink4a^/p19^Arf^ expression, reducing the potential for endothelial cell renewal and weakening the angiogenic abilities [33].

It was also attempted to determine the effect of miR-21 on micronuclide endothelial cells and the heart in acute myocardial infarction in rats. They showed that MiR-21 had a significant protective effect because it lowered both the size of the attack and the expression of the marker of damage by increasing the expression of VEGF and inhibiting the expression of PTEN in the rat model [34].

Chen and coauthors suggest that activation of the pathway STAT3/microRNA-21 is involved in angiotensin-induced angiogenesis and that inhibition of PTEN via STAT3 by the miR-21 induction in human microvascular endothelial cells (HMEC) exposed to Ang II is part of an epigenetic connector combining angiogenesis with atherosclerosis [35].

Acting in the heart fibroblasts, MiR-21 contributes to the reconstruction of myocardial infarction, which is a response to cardiac failure in the ischemia-reperfusion mechanism. In a report on the change in miR expression in response to myocardial ischemia-reperfusion (IR) in the heart of the mouse, it was shown that miR-21 regulates the expression of matrix metalloproteinase 2 (MMP-2) in cardiac fibroblasts in the infarction zone via the PTEN pathway [36].

MiR-21 also regulates the signalling pathway of ERK-MAP kinase in cardiac fibroblasts, which affects the overall structure and functions of the heart. The levels of miR-21 increase selectively in cardiac fibroblasts, increasing the activity of ERK-MAP kinase by inhibiting the homolog of sprouty1 (Spry1). This mechanism regulates the survival of fibroblasts and the secretion of growth factor, controlling interstitial fibrosis and cardiac hypertrophy [37]. Simultaneous inhibition of miR-21 expression and MAPK/ERK signalling inhibits the proliferation and fibrosis of the myocardium. This course of action appears to be an interesting therapeutic option for limiting myocardial fibrosis and even reversing left ventricular remodelling [38].

In recent years, a number of signalling pathways involved in the pathophysiological fibrosis process have been identified for miR-21, including the TGF-*β*/Smads, the 3-phosphatidyloinositol kinase (PI3K)/AKT, and the ERK/MAPK mitogen-activated kinase pathways [39].

MicroRNA-21 also acts as a cellular connector. Sayed and coauthors showed that miR-21 regulates the formation of branching and the cardiomyocyte outgrowths as a result of reducing the expression of SPRY2, which is an inhibitor of this process. The outgrowths connect adjacent cells through slotted joints. In addition, they concluded that the stimulation of *β*-adrenergic receptors leads to the upregulation of the miR-21, thereby contributing to the downregulation of SPRY2 and consequently to the formation of cellular branching. They suggested that this is an adaptive effect observed during myocardial hypertrophy when there is an increased expression of miR-21 associated with the redevelopment of the slotted joint and increased conduction rate. They also suggest that this effect is reversed during heart failure when the miR-21 level falls [40].

Scientific reports show that miR-21 is also an antagonizer of heart cell apoptosis. Its antiapoptotic role in the course of damage to reactive cardiomyocytes (ROS) by the PDCD4 gene was described by Cheng and others. They suggested that miR-21 could play a pivotal role in heart diseases associated with reactive oxygen species such as cardiac hypertrophy, cardiac failure, myocardial infarction, and ischemia [41]. MiR-21 also acts as an ischaotic-induced apoptotic cell by target binding to the gene programmed cell death (PDCD4) and the AP-1 activator protein pathway [42]. In addition, the overexpression of miR-21 in cardiomyocytes damaged by reactive oxygen forms protects cardiomyocytes against apoptosis. They identified the pivotal role of miR-21 modulation via NF-*κ*B in H_2_O_2_-induced oxidative stress in cardiomyocytes by aiming at PDCD4 [43]. In the research area, the antiapoptotic function of miR-21 was also used in exosomes from cardiac progenitor cells. The studies have confirmed that the exosomal miR-21 prevents the apoptosis of cardiomyocytes by aiming at PDCD4 [44]. A PTEN/Akt-dependent mechanism was also described, where miR-21 inhibits cardiomyocyte apoptosis—induced by ischemia-reperfusion and non-oxidation-reperfusion [45]. We know in detail the pathway in which miR-21 decreases apoptosis-induced hydrogen peroxide in cardiac stem cells in vitro by reducing the PTEN/PI3K/Akt signalling pathway and that miR-21 lowers the caspase-3 proapoptotic protein level and Bax and increases Bcl-2 [46].

The protective role of miR-21 in endothelial cells was also described by Zeng and others. Their studies have shown that miR-21 protects the endothelial cell against high glucose-induced endothelial cytotoxicity, possibly by inhibiting the expression of DAXX, which was considered an important step towards a better understanding of vascular diseases associated with diabetes mellitus [47].

One of the most recent discoveries is the demonstration of the role of miR-21 in reducing inflammation, dysfunction, and myocardial remodelling after myocardial infarction (MI) by aiming at KBTBD7 and inhibiting the activation of signalling p38 and NF-*κ*B, which may suggest that miR-21 may be considered a potential therapeutic target in the early stages of MI [48].

## 2. Discussion and Conclusions

Previous scientific reports confirm the specific role of microRNA-21 in the cardiovascular system. MiR-21 is expressed in cardiomyocytes, fibroblasts, and endothelial cells where apoptosis is adequately regulated, as well as fibrosis, proliferation, and cellular migration [49]. Acting so extensively and regulating numerous routes, the circulating miRNA-21 can be useful as a diagnostic/prognostic biomarker and as a therapeutic target in various cardiovascular diseases, especially in heart failure [50]. For example, during myocardial ischemia, the level of miR-21 decreases sharply, and supplementing its expression reduces the size of the attack and delays the development of HF [51]. It was also proposed that miR-21 could be an indicator of late age in ischemic heart failure [52]. Numerous studies indicate that both the heart and circulatory miR-21 correlate with the degree of cardiac fibrosis [53]. In systolic heart failure, microRNA-21 inhibits cardiac fibroblast apoptosis, which leads to hypertrophy and fibrosis of the myocardium [54]. In addition, miR-21 serum concentrations strongly positively correlate with the concentrations of galectin-3, which confirms the postulated role of miR-21 in fibrosis and makes miR-21 an interesting therapeutic target in heart failure [55]. MiR-21 was also considered a potential significant prognostic biomarker in patients with symptomatic heart failure and left ventricular hypertrophy [56]. In a short announcement, Tomaniak and coauthors concluded that patients with decompensated systolic heart failure had a higher miR-21 expression, but it was comparable regardless of the left ventricular dimension. “The expression of miR-21 was comparable regardless of LV size” [57]. Studies of cardiac injuries and disturbances caused by aldosterone in rats also suggest that the reduction in miR-21 expression intensifies cardiac hypertrophy, gene expression of fibrosis markers, interstitial fibrosis and perivascular disorders, and cardiac dysfunction [58]. However, Patrick and coauthors in studies on the mouse model noticed that inhibition of the expression miR-21 does not block the reconstruction of the heart in response to various cardiac stresses, and mice lacking the mir-21 gene still exhibit cardiac hypertrophy, fibrosis, regulation of heart genes, and loss of heart contractility [59]. Studies conducted by Qiao and coauthors in cell-based heart disease patients also indicate that miR-21 does not promote cardiac fibrosis as miR-21 regulates the signalling pathway of PTEN/act in cardiomyocytes and endothelial cells instead of fibroblasts, and additionally, through the exosome, it contributes to the repair of the heart by increasing the angiogenesis and survival of cardiomyocytes due to inhibition of apoptosis [60].

In conclusion, microRNA-21 can be a potential auxiliary biomarker, both diagnostic and prognostic, in cardiovascular diseases. It can also provide an interesting therapeutic target by limiting the progression of heart failure. However, discovering numerous and increasingly complex routes governed by miR-21 causes some limitations before the introduction of this and other microRNAs into the clinical field. In addition, most studies were conducted on small to moderate groups of cases. There are contrasting results by possible interfering factors, different methodologies, age of patients, presence of concomitant diseases, or actions of other microRNAs involved in cardiovascular mechanisms. There is therefore a need for further research including the definition of a comprehensive profile of microRNA expression and its correlation with the various pathways underlying the pathology of the cardiovascular system. However, it is certain that miR-21 plays a huge role in the cardiovascular system, which prompts further research to confirm the possibility of using the knowledge gathered in clinical practice.
